# Evaluation of a New Handheld Instrument for the Detection of Counterfeit Artesunate by Visual Fluorescence Comparison

**DOI:** 10.4269/ajtmh.13-0644

**Published:** 2014-11-05

**Authors:** Nicola Ranieri, Patricia Tabernero, Michael D. Green, Leigh Verbois, James Herrington, Eric Sampson, R. Duane Satzger, Chindaphone Phonlavong, Khamxay Thao, Paul N. Newton, Mark R. Witkowski

**Affiliations:** Forensic Chemistry Center, US Food and Drug Administration, Cincinnati, Ohio; Lao-Oxford-Mahosot Hospital-Wellcome Trust Research Unit (LOMWRU), Microbiology Laboratory, Mahosot Hospital, Vientiane, Lao People's Democratic Republic; Worldwide Antimalarial Resistance Network (WWARN), Nuffield Department of Clinical Medicine, University of Oxford, Oxford, United Kingdom; Centre for Tropical Medicine and Global Health, Nuffield Department of Clinical Medicine, Churchill Hospital, University of Oxford, Oxford, United Kingdom; Division of Parasitic Diseases and Malaria, Center for Disease Control, Atlanta, Georgia; Office of International Programs, Office of Global Regulatory Operations and Policy, US Food and Drug Administration, Silver Spring, Maryland; Fogarty International Center, National Institutes of Health, Bethesda, Maryland; Bureau of Food and Drug Inspection (BFDI), Ministry of Health, Government of the Lao People's Democratic Republic, Vientiane, Lao People's Democratic Republic; Food and Drug Quality Control Centre (FDQCC), Ministry of Health, Government of the Lao People's Democratic Republic, Vientiane, Lao People's Democratic Republic

## Abstract

There is an urgent need for accurate and inexpensive handheld instruments for the evaluation of medicine quality in the field. A blinded evaluation of the diagnostic accuracy of the Counterfeit Detection Device 3 (CD-3), developed by the US Food and Drug Administration Forensic Chemistry Center, was conducted in the Lao People's Democratic Republic. Two hundred three samples of the oral antimalarial artesunate were compared with authentic products using the CD-3 by a trainer and two trainees. The specificity (95% confidence interval [95% CI]), sensitivity (95% CI), positive predictive value (95% CI), and negative predictive value (95% CI) of the CD-3 for detecting counterfeit (falsified) artesunate were 100% (93.8–100%), 98.4% (93.8–99.7%), 100% (96.2–100%), and 97.4% (90.2–99.6%), respectively. Interobserver agreement for 203 samples of artesunate was 100%. The CD-3 holds promise as a relatively inexpensive and easy to use instrument for field evaluation of medicines, potentially empowering drug inspectors, customs agents, and pharmacists.

## Introduction

The World Health Organization (WHO) considers counterfeit or falsified (spurious/falsely labeled/falsified/counterfeit [SFFC]) medicines to be a major threat to public health, with counterfeit/falsified and substandard antimalarial pharmaceuticals being a major global public health concern, especially in areas of sub-Saharan Africa and southeast Asia.^1^–^3^ A very large epidemic of counterfeit artesunate afflicted mainland southeast Asia over the last decade.^4^–^8^ In Africa, there are increasing reports of poor artemisinin-based combination therapies (ACTs), which are the vital mainstay for malaria control.^5^,^9^–^11^ Widespread problems have also been reported for other essential medicines, such as antihelminthics, antibiotics, and antiretrovirals.^8^,^12^–^15^

Analysis of the quality of medicines poses severe problems, because few countries have WHO prequalified reference laboratories for chemical analysis. Three countries in malarious Africa and five countries in malarious Asia have such laboratories, and they are all in capital cities. Such analysis is expensive and requires significant human capacity, sustainable funding, and quality assurance.^16^ The WHO estimated that 30% of countries have either no medicine regulation in place or a very limited capacity that hardly functions.^17^,^18^ Considerable investment is, therefore, needed in building appropriate Medicine Regulatory Authority (MRA) capacity, including the capability for screening and analyzing medicines.^9^

Although development of centralized laboratories is important, it is also vital to implement analytical tools in the field in warehouses, at border crossings, and in pharmacies, empowering medicine inspectors to screen for medicines that require additional laboratory assessment. Rapid colorimetric tests and the Minilab for thin-layer chromatography analysis have been developed with this implementation in mind.^19^,^20^ In addition, many portable instruments are marketed for chemical analysis of medicines, which are based primarily on near-infrared (NIR) and Raman spectroscopy.^21^–^25^ However, such instruments may not be quantitative or suitable for all pharmaceutical products, are costly, and require sophisticated software and training. In low- and middle-income countries (L/MICs) in Africa and southeast Asia, rugged, low-cost, easy to use, validated field instruments are needed as screening tools to detect suspect counterfeit and substandard medicines for additional analysis.

Although the details are still debated, there are two main types of poor quality medicines: substandard and counterfeit (the latter is increasingly referred to as falsified in the global arena when intellectual property considerations are not invoked).^26^ In essence, substandard medicines are produced by authorized manufacturers, but because of errors in production and quality control, they fail to meet required pharmaceutical standards. In contrast, counterfeit or falsified medicines are fraudulent products manufactured with the intent to deceive. The term counterfeit, used by the Food and Drug Administration (FDA) and defined in the US Federal Food Drug and Cosmetic Act,^27^ applies to a product that falsely bears the trademark, trade name, or other identifying feature and thereby, purports to be an authentic product.

A medicine's packaging provides valuable points of comparison, and detection of differences from known authentic packaging for a given product in addition to chemical analysis are key for the detection of counterfeits. Counterfeit medicines may contain the active pharmaceutical ingredients (APIs), but when present, they are frequently in subtherapeutic quantities.^5^,^6^,^28^ Therefore, an instrument that enables rapid comparison of packaging and product may provide an accurate and less expensive tool for screening suspect medicines. If validated, the Counterfeit Detection Device version 3 (CD-3) combined with field-deployable spectroscopic tools and the Minilab may provide sufficient pharmaceutical quality information to make acceptance/rejection decisions in the field. Over the last 7 years, FDA scientists at the Forensic Chemistry Center (FCC) have developed and deployed several generations of this inexpensive handheld field instrument that can rapidly analyze medicines in real time, allowing onsite/port of entry real-time comparison of the pharmaceutical dosage form and the packaging with authentic examples.^29^ The current version, the CD-3, has been used routinely to screen suspect pharmaceutical products both at the FCC laboratory and in the field in the United States at border crossings and other points of entry. At the international mail facilities (IMFs) in the United States, the instruments are used by FDA investigators to screen incoming packages for potential counterfeit pharmaceuticals, cosmetics, and medical devices. The CD-3 has also been successfully used in a number of criminal investigations and has screened numerous suspect products in both the laboratory and the field. The CD-3 has been validated and used in the detection of counterfeits of pharmaceutical products commonly imported into the United States (e.g., Lipitor, Plavix, Viagra, and Cialis; FDA, unpublished data). However, no testing had been conducted on antimalarials, and the CD-3 has not been used outside of the United States, where counterfeit and substandard medicines are known to be significant problems. Therefore, the CD-3 was evaluated in the Lao People's Democratic Republic (Laos) with two objectives: to estimate the accuracy of the CD-3 in distinguishing counterfeit from genuine antimalarials as a proof of principle and to determine the interobserver variability in CD-3 interpretation among Laos drug inspectors after brief training on the use of the device.

## CD-3 Description

**Figure 1.**
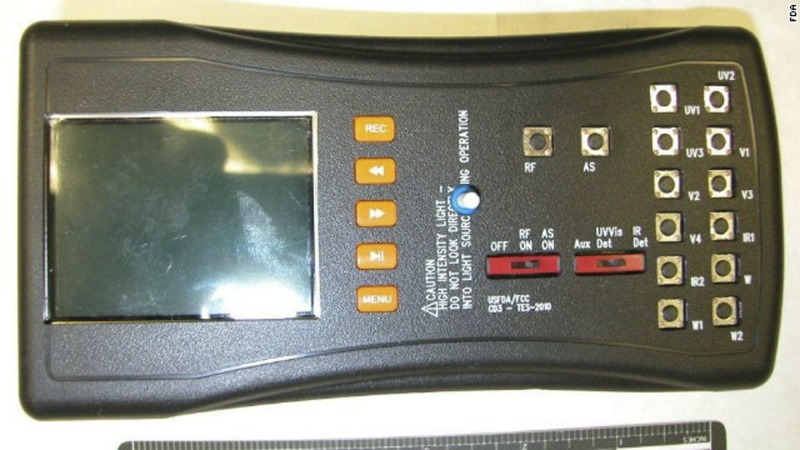
The CD-3 current generation of the instrument.

**Figure 2.**
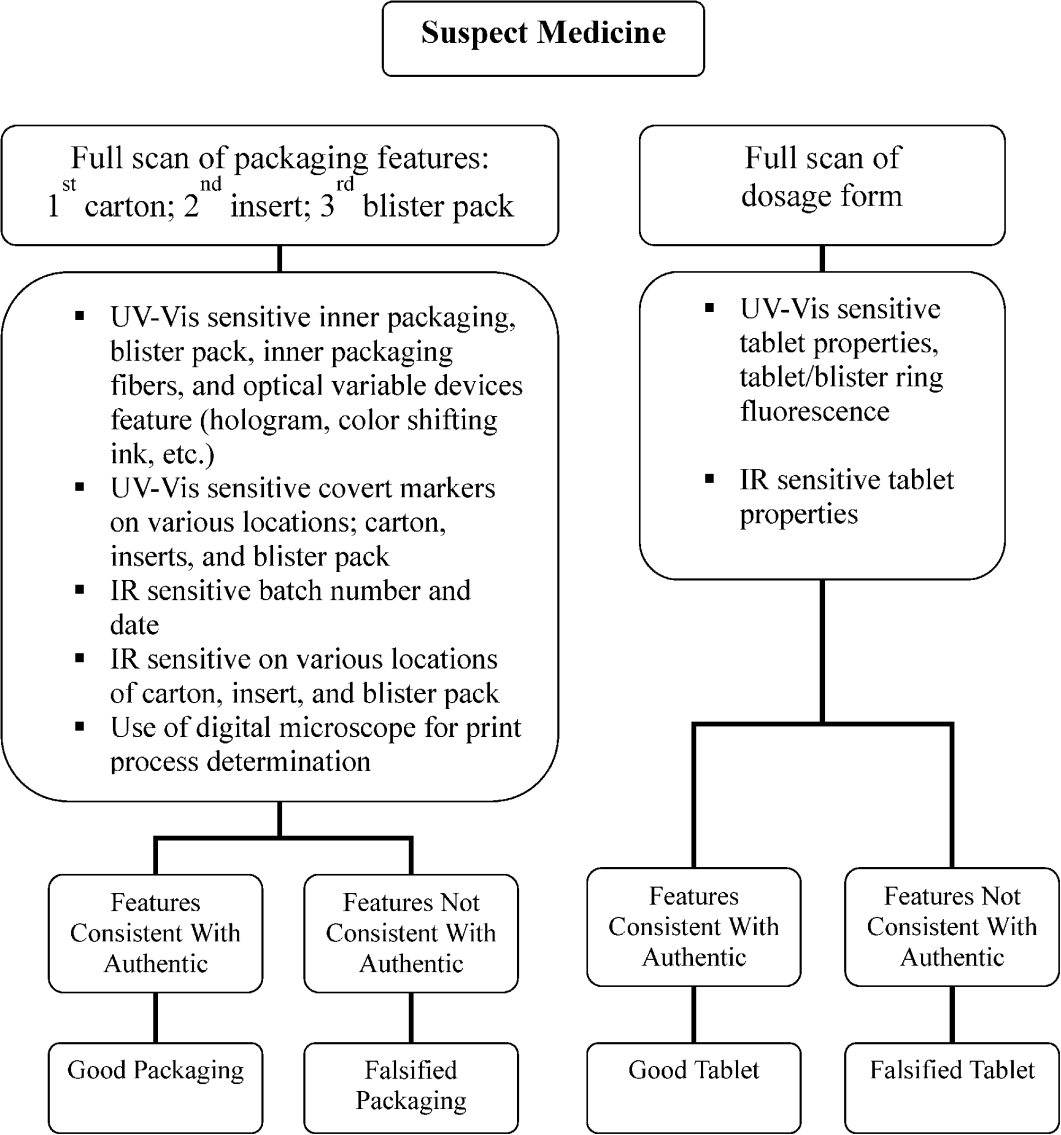
The testing algorithm used for suspect artesunate tablets.

The CD-3 is a compact (15.2 × 7.6 cm; 300 g) handheld electronic tool (Figure 1) with multiple single wavelength light-emitting diode (LED) light sources that cover the spectral region from the ultraviolet (UV) to the infrared (IR).^29^ The CD-3 features two charged couple device (CCD) cameras that provide the user with the ability to view samples in real time and capture images and videos of the suspect samples being screened. One camera operates in the UV-visible (UV-Vis) spectral region, and the second camera operates in the IR spectral region. It can be battery or mains powered, and a separate digital handheld microscope can be used to examine suspect samples at higher magnifications. The batteries will last between 3 and 8 hours depending on the intensity of use. The LEDs are used to illuminate a sample at user-selected wavelengths (375 and 470 nm) and enable the user to directly visualize product differences using either CCD camera. To put it in terms of a traditional spectrometer (e.g., NIR spectroscope), the LEDs are the light source, and the user's eye is the CD-3 detector. No *x*–*y* spectral data are generated or saved with the CD-3. The light from the LEDs interacts with the inks and tablet colors on sample packaging and dosage form surfaces. What the user's eye observes are differences between the suspect dosage form and packaging and an authentic drug. The visual differences observed can take the form of changes in colors, shading, contrast, fluorescence, or a combination of all of these changes. The CD-3 can be used (Figure 2) to verify the presence or absence of overt/covert markings on the packaging. The differences observed in the suspect sample can be attributed to chemical differences between the products (e.g., differences in active ingredient and excipients), colors used in the coatings, or packaging materials (e.g., cellulose materials used in boxes and outserts, inks, print type, etc.). Differences between suspect and authentic dosage forms are readily observed through blister packages, facilitating rapid product screening. The greater the number of visual differences observed using the CD-3 in a suspect sample compared with an authentic sample, the stronger the evidence that the product is counterfeit. The device captures jpeg images at 96 dots per inch (DPI) resolution. Dimensions of each captured image are 720 × 576 pixels (width × length) at 24-bit color depth. At these parameters, each captured image averages a file size of approximately 75 Kb. The device storage is an 8-GB (upgradable to 32 GB) flash drive that can hold approximately 107,000 CD-3 images. The drive is accessible using a personal computer through a Universal Serial Bus (USB) cable connection. Files can be uploaded to the drive or downloaded from the drive.

## Methods

All work was conducted at the Microbiology Laboratory, Mahosot Hospital, Vientiane, Laos in July of 2012. The samples were from a library of counterfeit and genuine antimalarials from different manufacturers collected in surveys over the last 10 years (described in refs. 4, 6, and 9 and unpublished data by Newton PN and Tabernero P) and stored at approximately 4°C. All samples had undergone visual packaging analysis with reference to known authentic drugs obtained directly from manufacturers as well as high-performance liquid chromatography (HPLC), mass spectrometry, or Fast Red Dye test assay.^19^ Counterfeit samples were defined as those failing packaging analysis, and genuine samples were those that passed both packaging and chemical analysis. No samples passed packaging analysis but failed chemical analysis. Because the authentic products had not been analyzed before using the CD-3, a baseline description or library of visual features was created for each authentic antimalarial product (packaging and dosage form) using all of the different CD-3 instrument settings (illumination wavelengths) for two authentic samples and entered into an Excel spreadsheet. The measurements of multiple unique markers for each authentic sample helped resolve any possible discrepancies when examining the suspect samples. Images for each CD-3 setting and responses were documented. The differences observed between genuine and counterfeit packaging are considered law enforcement-sensitive, and therefore, additional details of those observations are not provided.

Three individuals (a pharmacist and two drug inspectors) were initially trained in the use of the CD-3 for 1.5 days. The CD-3 users were then blinded to the authenticity of the specimens until after testing was completed (Figure 2). Interobserver variability was assessed for 102 samples. Over a 3-day period, 203 samples of oral antimalarial artesunate were scanned using the CD-3.

## Results

### Evaluation of the CD-3.

**Figure 3.**
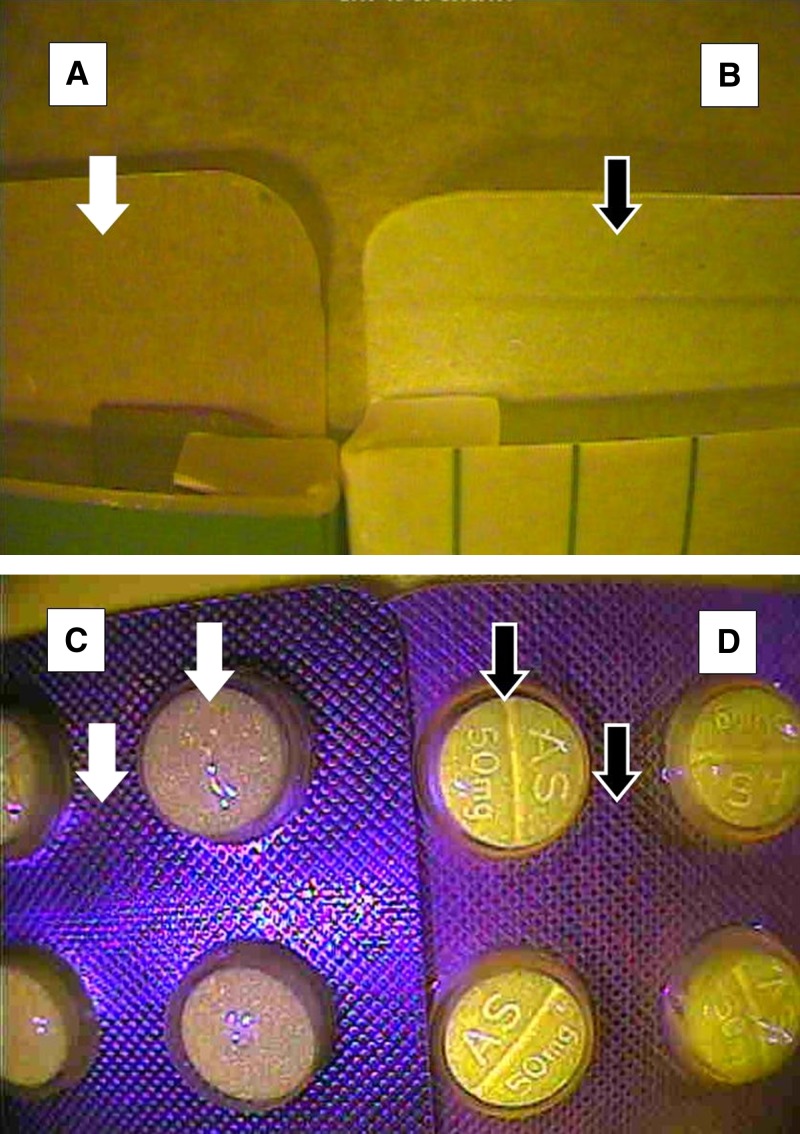
Examples of differences observed between counterfeit and authentic artesunate tablets and packaging. In general, clear differences between the suspect and the authentic tablets and packaging and many of the comparison features could not be detected with the naked eye. **A** and **B** show the interior of the cardboard box dark versus bright (regions are highlighted by white and black arrows, respectively); differences were observed between the (**A**) counterfeit and (**B**) authentic carton end flap using a 375-nm wavelength setting on the CD-3. **C** and **D** show the (**C**) absence of tablet debossing details for tablets in AS 50 mg and (**D**) enhanced tablet debossing details on AS 50 mg. Differences in tablet color and blister pack surface patterns (regions highlighted by white and black arrows, respectively) were observed between the (**C**) counterfeit and (**D**) authentic tablets in blisters using a 470-nm wavelength setting on the CD-3.

Two hundred three samples of artesunate tablet blister packs, labeled as manufactured by Guilin Pharmaceutical Co. Ltd. (Guilin, People's Republic of China) with expiration dates ranging between 2001 and 2011, were analyzed. Among the counterfeit artesunate, 14 different counterfeit packaging types were included.^6^ Only two samples were misclassified—two authentic drugs as defined by packaging and chemical analysis were incorrectly classified as counterfeit by interpretation of CD-3 images (Table 1). The identification of the counterfeit samples was based on the visual appearance of the dosage form and the packaging. In one case, the blister pack was missing (and all tablets had been used in the analysis), and only the box was evaluated; the other sample had different print features from the authentic sample on the expiration and manufacturing information. The specificity (95% confidence interval [95% CI]), sensitivity (95% CI), positive predictive value (95% CI), and negative predictive value (95% CI) of the CD-3 for detecting counterfeit artesunate were 100% (93.8–100%), 98.4% (93.8–99.7%), 100% (96.2–100%), and 97.4% (90.2–99.6%), respectively (Table 1). Examples of key non-covert features revealed by the CD-3 used to identify counterfeit artesunate are shown in Figure 3. Additional CD-3 evaluation of a small collection of other products (artemether-lumefantrine, dihydroartemisinin-piperaquine, and sulphamethopyrazine-pyrimethamine) showed clear differences in the packaging and dosage form of authentic and counterfeit products that were not apparent to the naked eye (data not presented).

### Interobserver variability.

The interobserver agreement between the trainer and the two trainees for 102 samples of artesunate blisters was 100% (κ = 1.0).

## Discussion and Conclusions

The CD-3 showed very good accuracy in detecting counterfeit artesunate packaging and tablets, which were fraudulently labeled as manufactured by one company. Minimal training was required, and agreement between the trainer and two trainees was 100%. The availability of authentic samples, provided by the manufacturer, is necessary for comparison with suspect samples. The CD-3 can store an image library of authentic products relevant to the sampling planned and the area involved. Cooperation of the private sector for prompt and secure delivery of authentic examples of their products to MRAs will be important for the accurate use of the CD-3 to establish and maintain up to date image libraries of each product on the CD-3, particularly in developing economic and regulatory environments, where product registration may not occur. Such libraries could be easily shared around the world to MRAs that use the CD-3. However, information on covert markers as well as CD-3 baseline markers will need to be carefully guarded. Furthermore, risk analysis will be needed to minimize escalation of the sophistication and use of counterfeit packaging, which has appeared to happen with artesunate,^6^ making the task of detecting counterfeit medicines progressively harder. Although this may result in increased sophistication of counterfeiting by criminals, increasing their costs and reducing illicit profit margins, it may also make it more difficult for MRAs to detect counterfeit products in the supply chain and warn patients and pharmacists.

There are several limitations to this study. Although the CD-3 is routinely used for numerous medicine types, we only evaluated it with one medicine type in large numbers; also, interobserver variability only involved two trainees, and a collection of authentic and suspect samples was used rather than a prospective, real-life assessment. We used artesunate oral monotherapy, because we had access to a collection of falsified samples to test the CD-3 as a proof of concept. However, because artesunate monotherapy is no longer the WHO recommended therapy to treat malaria, additional field evaluation with diverse genuine, substandard, and falsified ACTs is required.^7^ Additionally, more method development with the CD-3 is needed to determine its ability to detect substandard medicines, such as products with reduced amounts of active pharmaceutical ingredients that have been negligently manufactured by authorized manufacturers. Although not the strength of this device, these products may be detected if the manufacturing errors lead to visual differences on the exterior or interior surfaces of the tablet compared with good quality authentic products.

These initial validation results suggest that the CD-3 could be a useful tool; it requires minimal training and can rapidly screen large numbers of medicines in the field for counterfeits in L/MICs, such as Laos. The device is not yet commercially available but likely to cost considerably less than portable chemical analysis instruments, such as Raman or NIR. The CD-3 does not directly analyze the chemistry of the sample, unlike Raman and NIR, which produce interpretable chemical signatures of the tablet constituents. However, it does, through allowing the observer to rapidly recognize visible differences from the genuine samples, facilitate identification of abnormalities in the packaging and tablets. Additional evaluations with other medicines, both falsified and substandard, and in other geographical settings are required. Evaluation of the accuracy and field robustness of the CD-3 compared and combined with portable chemical analysis instruments would help clarify the roles of each modality.

## Figures and Tables

**Table 1:** Comparison between reference assays and CD-3 test results for detection of counterfeit artesunate among 203 samples of oral artesunate labeled as manufactured by Guilin Pharmaceutical Co. Ltd., Guilin, China

	Reference counterfeit	Reference genuine	Total
CD-3 counterfeit	125	0	125
CD-3 genuine	2	76	78
Total	127	76	203

## References

[1] World Health Organization (2011). World Health Organization Substandard/Spurious/Falsely-Labelled/Falsified/Counterfeit Medical Products. Sixty-Fourth World Health Assembly A64/16. Provisional Agenda Item 13.7. 2011.

[2] Nayyar GN, Breman JG, Newton PN, Herrington JE (2012). Poor quality antimalarial drugs in southeast Asia and sub-Saharan Africa. Lancet Infect Dis.

[3] Reddy D, Banerji J (2012). Commentary: counterfeit antimalarial drugs. Lancet Infect Dis.

[4] Newton PN, Proux S, Green M, Green M, Smithuis F, Rozendaal J, Prakongpan S, Chotivanich K, Mayxay M, Looareesuwan S, Farrar J, Nosten F, White NJ (2001). Fake artesunate in southeast Asia. Lancet.

[5] Newton PN, McGready R, Fernández F, Green MD, Sunjio M, Bruneton C, Phanouvong S, Millet P, Whitty CJ, Talisuna AO, Proux S, Christophel EM, Malenga G, Singhasivanon P, Bojang K, Kaur H, Palmer K, Day NPJ, Greenwood BM, Nosten F, White NJ (2006). Manslaughter by fake artesunate in Asia—will Africa be next?. PLoS Med.

[6] Newton PN, Fernández FM, Plançon A, Plançon A, Mildenhall DC, Green MD, Ziyong L, Christophel EM, Phanouvong S, Howells S, McIntosh E, Laurin P, Blum N, Hampton CY, Faure K, Nyadong L, Soong SWR, Santoso B, Zhiguang W, Newton J, Palmer K (2008). A collaborative epidemiological investigation into the criminal fake artesunate trade in south east Asia. PLoS Med.

[7] Sengaloundeth S, Green MD, Fernández FM, Manolin O, Phommavong K, Insixiengmay V, Hampton CY, Nyadong L, Mildenhall DC, Hostetler D, Khounsaknalath L, Vongsack L, Phompida S, Vanisaveth V, Syhakhang L, Newton PN (2009). A stratified random survey of the proportion of poor quality oral artesunate sold at medicine outlets in the Lao PDR—implications for therapeutic failure and drug resistance. Malar J.

[8] Lon CT, Tsuyuoka R, Phanouvong S, Nivanna N, Socheat D, Sokhan C, Blum N, Christophel EM, Smine A (2006). Counterfeit and substandard antimalarial drugs in Cambodia. Trans R Soc Trop Med Hyg.

[9] Newton PN, Green MD, Mildenhall DC, Plançon A, Nettey H, Nyadong L, Hostetler DM, Swamidoss I, Harris GA, Powell K, Timmermans AE, Amin AA, Opuni SK, Barbereau S, Faurant C, Soong RCW, Faure K, Thevanayagam J, Fernandes P, Kaur H, Angus B, Stepniewska K, Guerin PJ, Fernandez FM (2011). Poor quality vital anti-malarials in Africa—an urgent neglected public health priority. Malar J.

[10] WHO (2011). Survey of the Quality of Selected Antimalarial Medicines Circulating in Six Countries of Sub-Saharan Africa, 2011.

[11] WWARN Antimalarial Quality Surveyor.

[12] Khan MH, Okumura J, Sovannarith T, Nivanna N, Nagai H, Taga M, Yoshida N, Akazawa M, Tanimoto T, Kimura K (2011). Counterfeit medicines in Cambodia—possible causes. Pharm Res.

[13] Laing R, Vrakking H, Fourie B (2004). Quality and stability of TB medicines: let the buyer beware!. Int J Tuberc Lung Dis.

[14] Taylor RB, Shakoor O, Behrens A, Everard M, Low AS, Wangboonskul J, Reid RG, Kolawole JA (2001). Pharmacopoeial quality of drugs supplied by Nigerian pharmacies. Lancet.

[15] Amon JJ (2008). Dangerous medicines: unproven AIDS cures and counterfeit antiretroviral drugs. Global Health.

[16] World Health Organization (2014). WHO List of Prequalified Quality Control Laboratories.

[17] World Health Organization (2005). Counterfeit and Substandard Drugs. Frequently Asked Questions.

[18] Green MD, Mount DL, Wirtz RA (2001). Authentication of artemether, artesunate and dihydroartemisinin antimalarial tablets using a simple colorimetric method. Trop Med Int Health.

[19] Jähnke RWO, Pachaly P, Gobina NP, Schuster A, Nigge OJ, Dwornik K, Rubeau V, Davydova N, Bradby S, Hajjou M, Smine A, Phanouvong S (1998). Concise Quality Control Guide on Essential Drugs, Vol. II, Thin Layer Chromatography, Frankfurt: German Pharma Health Fund, 1998. Supplement 1999, 2002, 2003, and 2004.

[20] Dowell FE, Maghirang EB, Fernández FM, Newton PN, Green MD (2008). Detecting counterfeit antimalarial tablets by near-infrared spectroscopy. J Pharm Biomed Anal.

[21] Fernández FM, Hostetler D, Powell K, Kaur H, Green MD, Mildenhall DC, Newton PN (2011). Poor quality drugs: grand challenges in high throughput detection, countrywide sampling, and forensics in developing countries. Analyst (Lond).

[22] Ricci C, Eliasson C, Macleod NA, Newton PN, Matousek P, Kazarian SG (2007). Characterization of genuine and fake artesunate anti-malarial tablets using Fourier transform infrared imaging and spatially offset Raman spectroscopy through blister packs. Anal Bioanal Chem.

[23] Ricci C, Nyadong L, Yang F, Fernandez FM, Brown CD, Newton PN, Kazarian SG (2008). Assessment of hand-held Raman instrumentation for in situ screening for potentially counterfeit artesunate antimalarial tablets by FT-Raman spectroscopy and direct ionization mass spectrometry. Anal Chim Acta.

[24] Eliasson C, Matousek P (2007). Noninvasive authentication of pharmaceutical products through packaging using spatially offset raman spectroscopy. Anal Chem.

[25] Newton PN, Amin A, Bird C, Passmore P, Dukes G, Tomson G, Simons B, Bate R, Guerin PJ, White NJ (2011). The primacy of public health considerations in defining poor quality medicines. *PLoS Med*. 8.

[26] Hall KA, Newton PN, Green MD, De Veij M, Vandenaabele P, Pizzanelli D, Mayfong M, Dondorp A, Fernández F (2006). Characterization of counterfeit artesunate antimalarial tablets from southeast Asia. Am J Trop Med Hyg.

[27] US Government 21 USC 301, section 201(g)(2); 21 USC 311, section 301 (i)(1-3). http://www.gpo.gov/fdsys/pkg/USCODE-2013-title21/html/USCODE-2013-title21-chap9-subchapIII.htm.

[28] Food US, Administration Drug (2010). US Patent Application No. 13/262,371 (filed March 31, 2010) and EP Patent Application No. 107284765.5 (filed March 31, 2010.

[29] US Food and Drug Administration (2013). CD-3: A New Tool in FDA's Fight Against Counterfeit Products.

